# Acceptability and Correlates of Primary and Secondary Prevention of Cervical Cancer among Medical Students in Southwest China: Implications for Cancer Education

**DOI:** 10.1371/journal.pone.0110353

**Published:** 2014-10-31

**Authors:** Xiong-Fei Pan, Zhi-Mei Zhao, Jing Sun, Feng Chen, Qing-Lian Wen, Kang Liu, Gui-Qin Song, Jing-Jing Zhang, Ying Wen, Chun-Jing Fu, Chun-Xia Yang

**Affiliations:** 1 Department of Epidemiology, West China School of Public Health, Sichuan University, Chengdu, China; 2 Department of Pathology, Development and Regeneration Key Laboratory of Sichuan Province, Chengdu Medical College, Chengdu, China; 3 Department of Preventive Healthcare and Hospital Infection Control, Deyang People's Hospital, Deyang, China; 4 Department of Oncology, the Affiliated Hospital of Luzhou Medical College, Luzhou, China; 5 Institute of Tissue Engineering and Stem Cells, The Second Clinical Hospital of North Sichuan Medical College, Nanchong, China; 6 Department of Biology, North Sichuan Medical University, Nanchong, China; 7 School of Public Health, Kunming Medical University, Kunming, China; 8 Department of Health Statistics and Information Management, School of Public Health and Management, Chongqing Medical University, Chongqing, China; National Institute for Viral Disease Control and Prevention, CDC, China, China

## Abstract

**Objectives:**

To understand knowledge about, and acceptability of, cervical cancer screening and HPV vaccines among medical students; and to explore potential factors that influence their acceptability in China.

**Methods:**

We conducted a survey among medical students at six universities across southwest China using a 58-item questionnaire regarding knowledge and perceptions of HPV, cervical cancer, and HPV vaccines.

**Results:**

We surveyed 1878 medical students with a mean age of 20.8 years (standard deviation: 1.3 years). Of these, 48.8% and 80.1% believed cervical cancer can be prevented by HPV vaccines and screening respectively, while 60.2% and 71.2% would like to receive or recommend HPV vaccines and screening. 35.4% thought HPV vaccines ought to be given to adolescents aged 13–18 years. 32% stated that women should start to undergo screening from the age of 25. 49.2% felt that women should receive screening every year. Concern about side effects (38.3% and 39.8%), and inadequate information (42.4% and 35.0%) were the most cited barriers to receiving or recommending HPV vaccination and cervical cancer screening. Females were more likely to accept HPV vaccines (OR, 1.86; 95% CI: 1.47–2.35) or cervical cancer screening (OR, 3.69; 95% CI: 2.88–4.74). Students with a higher level of related knowledge were much more willing to receive or recommend vaccines (*P*<0.001) or screening (*P*<0.001). Students who showed negative or uncertain attitudes towards premarital sex were less likely to accept either HPV vaccines (OR, 0.67; 95% CI: 0.47–0.96), or screening (OR, 0.68; 0.47–0.10). Non-clinical students showed lower acceptability of cervical screening compared to students in clinical medicine (OR, 0.74; 95% CI: 0.56–0.96).

**Conclusions:**

The acceptability of HPV vaccines and cervical cancer screening is relatively low among medical students in southwest China. Measures should be taken to improve knowledge about cervical cancer and awareness of HPV vaccines and screening among medical students at university.

## Introduction

Cervical cancer is the third most common malignancy among women, with an estimated 528,000 new cases and 266,000 deaths worldwide in 2012 [1]. In China, its age-standardized incidence and mortality rates of 7.5 and 3.4 per 100,000 women respectively are lower than corresponding world statistics (14.0 and 6.8 per 100,000). Given the large population of China, absolute estimates of cases and mortalities still make it one of the top priorities for cancer prevention and control.

Cervical cancer can be effectively controlled through primary and secondary prevention such as cervical screening and prophylactic HPV vaccination. Since the Papanicolaou (Pap) smear test was introduced for routine screening, a substantial decline has been witnessed in cervical cancer deaths in developed countries in the last four decades [2]. It is a general consensus that the cytology screening for cervical cancer is effective in reducing the incidence and mortality in developed countries. Visual inspection with acetic acid (VIA) or Lugol's iodine (VILI), and HPV DNA-based testing are also utilized for screening purposes in developing [3]–[5], and developed, countries [6], respectively. Prophylactic HPV vaccines have become an established and vital strategy for primary prevention of cervical cancer [7]. Gardasil quadrivalent HPV 6/11/16/18 vaccine developed by Merck (Whitehouse Station, New Jersey, United States) and Cervarix bivalent HPV 16/18 vaccine by GlaxoSmithKline (Brentford, London, United Kingdom) are widely available in over 100 countries through regional or national immunization programs. They had been introduced into national immunization programs in at least 40 countries by the beginning of 2012 [8]. The effectiveness and safety of these have been established in clinical trials and post-market surveillance in populations [9]–[12]. A systematic review of HPV vaccine clinical trials shows that efficacy is 50–90% in preventing intraepithelial neoplasm grade 2+ (CIN 2+) associated with HPV 16 and 18 [13]. Post-market experience also indicates that vaccine-type HPV prevalence in the US and the UK [14]–[16] and CIN 2+ incidence in Australia [9] decreased after the implementation of population-wide HPV vaccination programs.

However, health care providers have lagged behind in their efforts to improve screening for cervical cancer in China. There are no national guidelines for cervical cancer screening, though non-binding recommendations were made by the China Foundation of Cancer in collaboration with the Ministry of Health in 2009, that proposed cervical screening every 3 years for women between 25 and 65 years of age in urban areas, and between 35 and 65 years of age in rural areas [17]. There is no routinely organized national screening in the country. Since 2009 in rural China, free screening of cervical cancer has been available to a limited proportion of the target population in the form of government-sponsored mass screening [18]. An earlier large-scale free screening initiative based on visual inspection and cytology has only covered approximately10 million rural women between 35 and 59 years of age between 2009 and 2012 [19], while hopes have been expressed that ongoing efforts will be able to screen 50 million women in rural areas aged 35–64 years by 2015. Despite the increase in reported mortalities due to cervical cancer among young urban women at an annual rate of 4.1% [20], women in urban areas are referred to cervical cancer screening only on an opportunistic basis, or through employment-based physical examination [21]. In addition to limited coverage, the effectiveness of the cervical cancer screening program is hampered by the limited health care infrastructure available for the latest screening technologies, which also restrict attempts to promote the services nationally.

To date, the two prophylactic HPV vaccines available internationally are still to be approved by the China Food and Drug Administration, and are not commercially available in mainland China [19]. Clinical studies are underway among Chinese women: Gardasil and Cervarix have already been studied in trials for over four years, while a new HPV 16/18 vaccine by Xiamen Innovax Biotech (Xiamen, China) began the phase III clinical stage in 2013 [22]. An earlier systematic review showed that 69.7% of cervical cancer cases were attributed to HPV 16/18 in China [23], which is similar to the estimate (about 70%) at the global scale [24]. Given these estimates, the efficacy of HPV vaccines in Chinese trials may hopefully not be very much different from those from international studies [13]. In addition, estimated HPV 16/18 positive fractions in high-grade squamous intraepithelial lesion, low-grade squamous intraepithelial lesion, and normal women were 45.5%, 32.23% and 4.6%, respectively [23]. HPV vaccines are expected to effectively avert cervical morbidity and mortality in China if it was routinely used in women.

With the rapid scale-up of cervical cancer screening and anticipation of licensure for prophylactic HPV vaccines in China, it is important to understand acceptability of, and possible barriers to, screening and vaccination among the population. To date, previous studies have explored these issues among women, parents of adolescents, female university students, health care providers, and health management staff for HPV vaccines [25]–[28], and among the female part of the general population for cervical cancer screening [29], [30]. However, none of these studies have directly surveyed current medical students for their knowledge and perceptions, although this type of study has been conducted in other countries [31]–[35]. Given that these students will be future health care providers, their attitudes could influence the success of screening and vaccination programs. To fill the gap in the research, we systematically conducted a large-scale survey among medical students to better understand their knowledge, the acceptability of cervical cancer screening and HPV vaccines, and explore potential factors that influence acceptability.

## Materials and Methods

### 1. Study design and population

This cross-sectional study was conducted between May and September 2013 at six universities across southwest China, including Sichuan University and Chengdu Medical College in Chengdu, Luzhou Medical University in Luzhou, North Sichuan Medical College in Nanchong, Chongqing Medical University in Chongqing, and Kunming Medical University in Kunming. These universities are the principle institutions of medical education in three major provinces in southwest China. We planned to use 200–400 medical students from different years of study from each university as sample to ensure diversity. In addition, 5–6 whole classes of medical undergraduates were selected from clinical medicine and other non-clinical major subjects such as nursing, dentistry, public health, pre-clinical science, and medical technological science. Clinical medicine and non-clinical majors have different foci in China: clinical medicine and dentistry involve more clinical work, while other major subjects may focus on medical lab science, health management, public health, and other health related work. Due to financial and logistic constraints, the classes from each university were surveyed based on convenient contact points instead of a random sampling process. The study was approved by the Ethics Committee of Sichuan University Fourth Hospital/West China School of Public Health.

### 2. Study procedure

We used a 58-item questionnaire comprised of three sections of closed- and open-ended questions relating to basic information concerning participants, knowledge and perceptions of HPV, cervical cancer, HPV vaccines, cervical cancer screening, and sexual attitudes and behavior (Figure S1). The provisional questionnaire was formulated based on earlier ones used for similar studies in China [25]–[27] and abroad [33], [35] for survey purposes. It was revised according to comments solicited from colleagues who had administered similar surveys for earlier studies [25]–[27]. It was piloted by ten medical students in the Chinese Academy of Medical Sciences Cancer Institute, and changes were made to the questionnaire based on their comments regarding the appropriateness of contents and language. The pilot survey procedure was repeated on another ten medical students from Sichuan University. The questionnaire was finalized after internal discussions following the two pilot surveys.

Collaborating staff and medical students were trained on administering the questionnaire, and conducted the survey before or after class at each university between May and September 2013. Survey objectives were explained to potential participants before each survey. The medical students were free to participate, or not, at their discretion. The informed consent form was bound together with the questionnaire when it was handed out to them. Since the survey was anonymous to protect privacy and ensure data integrity, there was no request for a signature on the consent form if they agreed on the survey; they could choose to answer the questionnaire if they consented verbally or just leave it blank if they did not after reading the consent form. In accordance with the ethics committee document, no consent, verbal or written, was requested from parents or guardians of any participants. Our surveyors were responsive to queries about the questionnaire itself during the survey.

Completed questionnaires from each university were delivered to the Data Management Team in the West China School of Public Health. Data were entered and managed by the team in a database that had been developed by EpidData v3.1 (EpiData Association, Odense, Denmark).

### 3. Statistical analysis

IBM SPSS 19.0 (Armonk, New York, USA) was used to analyze the data. Demographic information, selected questions regarding HPV and cervical cancer related knowledge, and perceptions of cervical cancer and screening were presented as frequencies and percentages. Perceptions and concerns of primary and secondary prevention of cervical cancer were compared between males and females using the Chi-square test. Univariable logistic regression analysis was conducted to predict factors influencing the acceptability of cervical cancer and HPV vaccination, and the overall predictive model was established using multivariable logistic regression analysis based on factors of statistical significance. All tests were two-tailed with a significance level of 0.05.

## Results

### 1. Demographic characteristics

A total of 2150 medical students were approached, and 2000 successfully completed the questionnaires. However, another 122 questionnaires were discarded because they contained answers to only a few questions, or contained inconsistencies in the answers. Demographic characteristics of the final 1878 medical students are summarized in Table 1. These students consisted of 595 males (32.1%) and 1260 females (67.9%). The mean age was 20.8 (standard deviation: 1.3) years. 91.8% were of Han Chinese ethnicity, and 43.8% majored in clinical medicine. Most were second-year (36.2%) and third-year (37%) students. 85.1% of the students did not have clinical internship at the hospital. Regarding sexual attitude and behavior questions, 51.0% and 15.2%, respectively held neutral and positive attitude towards premarital sexual behavior, and 8.3% had previous experience of sexual activity.

**Table 1:** Demographic characteristics of medical students (N = 1878).

Characteristics	Frequency	Percentage
Age (years) ^a^		
17–19	260	14.2
20–22	1379	75.4
23–25	191	10.4
Gender ^b^		
Male	595	32.1
Female	1260	67.9
Ethnic groups^ c^		
Han	1697	91.8
Other	151	8.2
Grade		
1	243	12.9
2	679	36.2
3	695	37
4	204	10.9
5	57	3
Major		
Clinical	823	43.8
Non-clinical	1055	56.2
Clinical internship		
Yes	280	14.9
No	1598	85.1
Attitude towards premarital sexual behavior ^d^		
Positive	276	15.2
Negative	435	24
Neutral	927	51
Undecided	178	9.8
Previous sexual behavior^ e^		
Yes	150	8.3
No	1661	91.7

a,b,c,d,e Data were missing for 48, 23, 30, 62 and 67 students, respectively.

### 2. Knowledge of HPV, cervical cancer, HPV vaccines and cervical cancer screening

Responses to selected knowledge questions from the questionnaire are presented in Table 2. 76.5% of the medical students were aware of HPV, but only 29% knew there is an HPV vaccine available worldwide. Only 14.4% thought that persistent HPV infection was the necessary cause of cervical cancer. 47.8% thought that cervical cancer may be cured, while 48.8% and 80.1% believed that cervical cancer may be prevented by HPV vaccines or cervical screening. In addition, 72.6% agreed that women who had already been vaccinated require cervical screening. In general, higher percentages of women knew the correct answer to each selected knowledge question (Table 2).

**Table 2:** Summary of Related Knowledge Among Medical Students (N = 1878).

Questions*			Males (n = 595)		Females (n = 1260)		*P*
	Frequency	%	Frequency	%	Frequency	%	
Have you ever heard of HPV (yes)?^ a^	1436	76.5	441	74.1	982	77.9	0.07
Is persistent HPV infection the necessary cause of cervical cancer (yes)? ^b^	271	14.4	87	14.6	178	14.1	0.776
Can cervical cancer be cured (yes, only early cancer or precursor lesions)? ^c^	898	47.8	264	44.4	624	49.5	0.038
Can cervical cancer be prevented by HPV vaccines (yes)? ^d^	916	48.8	269	45.2	636	50.5	0.03
Is there any HPV vaccine available for cervical cancer, penile cancer, anal cancer, and genital warts, globally (yes)? ^e^	544	29	147	24.7	390	31	0.01
Can cervical cancer be prevented by screening (yes)? f	1485	80.1	422	72.5	1046	83.7	<0.001
Do women who have already been vaccinated require cervical cancer screening (yes)? ^g^	1364	72.6	376	63.2	976	77.5	<0.001

*The correct answer is stated in parentheses;

a, b, c, d, e, f, g Gender data were missing for 13, 6, 10, 11, 7, 17, and 12 students who gave a correct answer to each of these questions;

f 24 students did not answer this question.

### 3. Perceptions of HPV vaccination and cervical cancer screening

Perceptions of HPV vaccination among medical students are shown in Table 3. 60.2% of the medical students would like to receive or recommend HPV vaccination (49.3% for males versus 65.3% for females, *P*<0.001). 60.8% of female students though HPV vaccines could be given to boys, as compared to 50.0% of males students (*P*<0.001). 36.8% of male, and 34.6% of female, students preferred HPV vaccination for adolescents aged 13–18 years, and 63.5% and 66.0% of them thought that the best time for HPV vaccination would be before becoming sexually active. Most students stated that the local Center for Disease Prevention and Control (CDC) is the most appropriate venue for HPV vaccination, and the least selected venue was school (24.2% versus 22.2%). Over 70% of medical students were willing to pay a price lower than 500 RMB (82 USD) for either imported or domestic vaccines. In addition, 45.8% medical students expressed preference for imported vaccines, while 36.5% would like to make their choice according to the price.

**Table 3:** Perceptions of HPV vaccines among medical students (N = 1878).

			Males (n = 595)		Females (n = 1260)		*P*
Questions	Frequency	%	Frequency	%	Frequency	%	
Would you like to receive or recommend HPV vaccination? ^a^							<0.001
Yes	1128	60.2	293	49.3	821	65.3	
No/I don't know	747	39.8	301	50.7	437	34.7	
To which age group HPV vaccines should be given?^ b^							<0.001
0–12 years	191	10.2	94	15.8	92	7.3	
13–18 years	664	35.4	219	36.8	435	34.6	
19–25 years	526	28.0	113	19.0	409	32.5	
26 years & above	155	8.3	40	6.7	114	9.1	
Unknown	340	18.1	129	21.7	208	16.5	
Can HPV vaccines be given to boys?^ c^							<0.001
Yes	1072	57.4	296	50.0	763	60.8	
No	286	15.3	109	18.4	175	14.0	
Unknown	511	27.3	187	31.6	316	25.2	
Which is the most appropriate stage for HPV vaccination?^ d^							0.103
Before sexual debut	1219	65.1	376	63.5	830	66.0	
After sexual debut	181	9.7	50	8.4	128	10.2	
Unknown	472	25.2	166	28.0	299	23.8	
Which venue is the most appropriate for HPV vaccination (multiple responses)?^ e^							
Local CDC	1294	68.9	405	68.1	872	69.3	0.604
Community health center/local clinic	782	41.7	243	40.8	529	42.0	0.631
Women and children's hospital	1085	57.8	272	45.7	800	63.5	<0.001
General hospital	744	39.6	233	39.2	503	40.0	0.745
School	427	22.7	144	24.2	279	22.2	0.328
Unknown	58	3.1	24	4.0	34	2.7	0.124
How much are you willing to pay for imported HPV vaccines (RMB)?^ f^ #							<0.001
Below 100	547	29.2	232	39.1	306	24.4	
100–300	521	27.8	147	24.8	368	29.3	
300–500	398	21.3	107	18.0	286	22.8	
500–1000	284	15.2	70	11.8	213	17.0	
1000–2400	122	6.5	37	6.2	83	6.6	
How much are you willing to pay for domestic HPV vaccines (RMB)?^g^ #							<0.001
Below 100	754	40.3	289	48.8	455	36.2	
100–300	585	31.2	143	24.2	437	34.7	
300–500	285	15.2	84	14.2	198	15.7	
500–1000	200	10.7	58	9.8	138	11.0	
1000–2400	49	2.6	18	3.0	30	2.4	
Which vaccines you would like to receive or recommend?^ h^							0.187
Domestic HPV vaccines	331	17.7	108	18.2	214	17.1	
Imported HPV vaccines	858	45.8	255	42.9	594	47.4	
Either, dependent on price	683	36.5	232	39.0	446	35.6	

a, b, c, d, e, f, g, h 3, 2, 9, 6, 1, 6, 5 and 6 students, respectively, did not answer these questions, and gender data were missing for 23 students who answered these questions;

# 1 RMB equals to 0.16 US dollars.

Perceptions of cervical cancer screening among medical students are presented in Table 4. 71.2% of the medical students would like to receive or recommend cervical cancer screening (53.6% for males versus 79.4% for females, *P*<0.001). However, 45.2% and 39.6% of males and females were not aware of any existing screening techniques. 41.9% and 32% stated women ought to start being screened from 20 or 25 years, and 49.2% and 42.1% were of the opinion women should receive screening every year or every 2–4 years. 82.7% medical students thought a price below 100 RMB (16.5 USD) was reasonable for cervical cancer screening per occasion.

**Table 4:** Perceptions of cervical cancer screening among medical students (N = 1878).

			Males (n = 595)		Females (n = 1260)		*P*
Questions	Frequency	%	Frequency	%	Frequency	%	
Would you like to receive or recommend cervical cancer screening? a							<0.001
Yes	1311	71.2	308	53.6	988	79.4	
No/I don't know	530	28.8	267	46.5	257	20.6	
What techniques are you aware of for cervical cancer screening (multiple responses)? ^b^							
Pap smear	584	31.6	170	28.7	414	32.9	0.067
HPV DNA	761	41.1	226	38.1	535	42.5	0.072
VIA/VILI	491	26.5	134	22.6	357	28.4	0.009
Unknown	766	41.4	268	45.2	498	39.6	
When should women start screening? ^c^							<0.001
20 years	772	41.9	234	40	538	42.8	
25 years	589	32	154	26.3	435	34.6	
30 years	225	12.2	84	14.4	141	11.2	
35 years	108	5.9	45	7.7	63	5	
Unknown	124	6.7	60	10.3	64	5.1	
Others	23	1.2	8	1.4	15	1.2	
How often should women have cervical cancer screening?^ d^							0.104
Every 1 year	903	49.2	271	46.4	632	50.5	
Every 2–4 years	773	42.1	258	44.2	515	41.2	
Every 5–9 years	128	7	40	6.8	88	7	
Every over 10 years	31	1.7	15	2.6	16	1.3	
How much do you think is reasonable for cervical cancer screening for one occasion (RMB)?^ e^ #							<0.001
<10	285	15.5	139	24	146	11.6	
10–49	651	35.5	178	30.8	473	37.6	
50–99	581	31.7	159	27.5	422	33.6	
100–249	232	12.6	71	12.3	161	12.8	
150–199	74	4	22	3.8	52	4.1	
200 or above	12	0.7	9	1.6	3	0.2	

a, b, c, d, e 25, 4, 14, 20, and 20 students, respectively, did not answer these questions.

a gender data were missing for 22 students who answered these question;

b, c, d, e gender data were missing for 23 students who answered these questions;

# 1 RMB equals to 0.16 US dollars.

### 4. Concerns of HPV vaccination and cervical cancer screening

The concern about side effects (38.3% and 39.8%), and inadequate information (42.4% and 35.0%), were the obstacles most cited against receiving or recommending HPV vaccination and cervical cancer screening (Table 5). 81.5% of medical students favored a future HPV vaccination program in China, but about half of these would request pricing regulation and subsidy for recipients.

**Table 5:** Concerns of HPV vaccines and cervical cancer screening among medical students.

			Males		Females		*P*
Questions	Frequency	%	Frequency	%	Frequency	%	
What do you think will be the most important obstacle preventing yourself to receive or recommend HPV vaccination? a							0.002
High cost	35	10.1	17	10.8	18	9.8	
Concern about side effects	133	38.3	46	29.1	85	46.4	
Concern about efficacy of vaccines	32	9.2	11	7.0	18	9.8	
Inadequate information	147	42.4	84	53.2	62	33.9	
What is your attitude toward future HPV vaccination program in China (multiple responses)? b							
Positive, since the vaccines can prevent cervical cancer/genital warts	761	40.6	273	46.0	478	38.0	0.001
Positive, but requesting pricing regulation and subsidy	767	40.9	204	34.4	557	44.2	<0.001
Neutral, since the price will be high and the consumption capacity of ordinary Chinese should be considered.	152	8.1	67	11.3	81	6.4	<0.001
Neutral, since long-term efficacy and side effects should be evaluated.	276	14.7	77	13.0	194	15.4	0.168
Negative, since it may lead to promiscuity.	23	1.2	5	0.8	17	1.4	0.347
Other	17	0.9	8	1.3	9	0.7	0.182
What do you think will be the most important obstacle preventing yourself to receive or recommend cervical cancer screening?							0.123
High cost	53	16.9	30	17.4	23	16.2	
Concern about side effects	125	39.8	59	34.3	66	46.5	
Concern about effectiveness	26	8.3	14	8.1	12	8.5	
Inadequate information	110	35.0	69	40.1	41	28.9	

a Gender data were missing for 6 students who answered the question;

b 3 students did not answer the question, and gender data were missing for 23 students who answered these questions.

### 5. Correlates with acceptability of HPV vaccines and cervical cancer screening

Associations of acceptability of HPV vaccination and cervical cancer screening, with other factors, are presented in Table 6. In this research, the acceptability corresponds to the two questions on whether students would like to receive or recommend HPV vaccination or cervical cancer screening in Table 3 and 4. Six out of nine variables (eight variables in Table 1 and HPV related knowledge score) analyzed were significantly associated with the acceptability of either HPV vaccination or cervical cancer screening in the univariable logistic analysis, and were thus incorporated into the multivariable logistic modeling. Female students were more likely to accept HPV vaccines (OR, 1.86; 95% CI: 1.47–2.35) or cervical cancer screening (OR, 3.69; 95% CI: 2.88–4.74). Students who showed negative attitude towards premarital sex were less like to accept either HPV vaccines (OR, 0.67; 95% CI: 0.47–0.96) or screening (OR, 0.68; 0.47–0.10). Those who scored high on the level of relevant knowledge were much more willing to receive or recommend vaccination (*P*<0.001) or screening (*P*<0.001). In addition, non-clinical students showed lower acceptability of cervical screening compared to students in clinical medicine (OR, 0.74; 95% CI: 0.56–0.96). There were no statistically significant differences in acceptability of HPV vaccination or cervical cancer screening between students at different year groups (*P* = 0.218 and 0.091), and between students with or without clinical internship (*P* = 0.854 and 0.180). Moreover, non-clinical students did not express lower degree of likeliness to accept HPV vaccination (*P* = 0.376).

**Table 6:** Logistic regression analysis of acceptability of HPV vaccines and cervical cancer screening among medical students.

	Acceptability of vaccination (N = 1768)				Acceptance of cancer screening (N = 1740)		
	Subjects in analysis		n (%)	OR (95% CI)#	Subjects in analysis	n (%)	OR (95% CI)#
Gender							
Male		563	282 (50.1)	1	546	294 (53.8)	1
Female		1205	788 (65.4)	1.86 (1.47–2.35)	1194	952 (79.7)	3.69 (2.88–4.74)
				Overall *P*<0.001			Overall *P*<0.001
Grade							
1		216	120 (55.6)	1	209	143 (68.4)	1
2		636	345 (54.2)	0.68 (0.48–0.96)	622	424 (68.2)	0.76 (0.52–1.10)
3		665	443 (66.6)	0.76 (0.52–1.10)	660	481 (72.9)	0.60 (0.39–0.90)
4		195	126 (64.6)	0.80 (0.44–1.48)	193	154 (79.8)	0.92 (0.46–1.83)
5		56	36 (64.3)	0.57 (0.25–1.32)	56	44 (78.6)	0.57 (0.22–1.50)
				Overall *P* = 0.218			Overall *P* = 0.091
Major							
Clinical		765	487 (63.7)	1	751	549 (73.1)	1
Non-clinical		1003	583 (58.1)	0.90 (0.70–1.14)	989	697 (70.5)	0.74 (0.56–0.96)
				Overall *P* = 0.376			Overall *P* = 0.025
Clinical internship							
Yes		269	171 (63.6)	1	267	212 (79.4)	1
No		1499	899 (60.0)	0.95 (0.57–1.59)	1473	1034 (70.2)	0.67 (0.37–1.20)
				Overall *P* = 0.854			Overall *P* = 0.180
Attitude towards premarital sexual behavior							
Positive		264	154 (58.3)	1	260	170 (65.4)	1
Neutral		907	593 (65.4)	0.95 (0.69–1.30)	893	696 (77.9)	1.24 (0.89–1.74)
Negative		427	241 (56.4)	0.67 (0.47–0.96)	421	291 (69.1)	0.68 (0.47–1.00)
Uncertain		170	82 (48.2)	0.59 (0.38–0.91)	166	89 (53.6)	0.42 (0.27–0.66)
				Overall *P* = 0.006			Overall *P*<0.001
Level of related knowledge a							
0–1		238	58 (24.4)	1	232	95 (40.9)	1
2–3		507	248 (48.9)	2.86 (2.01–4.06)	499	344 (68.9)	2.94 (2.08–4.16)
4–5		736	530 (72.0)	7.50 (5.28–10.67)	726	569 (78.4)	4.59 (3.24–6.49)
6–7		287	234 (81.5)	12.80 (8.23–19.89)	283	238 (84.1)	6.90 (4.40–10.84)
				Overall *P*<0.001			Overall *P*<0.001

# adjusted for other variables in the table;

a The level of HPV related knowledge was represented by a summary score, and 1 score was given only if a right answer was given for one of the 7 selected questions in Table 2.

## Discussion

To our knowledge, this is the largest multicenter study that has explored the acceptability of HPV vaccines and cervical cancer screening among medical students in China. One major finding is that 60.2% and 71.2% of medical students were willing to accept or recommend HPV vaccines and cervical cancer screening, respectively. Female students and students with improved knowledge were positive predictors for both HPV vaccines and screening, while a negative attitude towards premarital sex was a negative correlate for both. In addition, non-clinical students were less likely to accept cervical cancer screening. We also noticed certain concerns and perceptions of these students regarding future HPV vaccines and screening programs. In particular, the concern about side-effects and inadequate information were the two most cited concerns connected with HPV vaccines and cervical cancer screening among medical students.

The acceptance of both primary and secondary prevention of cervical cancer was relatively low in our study. The acceptability of HPV vaccines was similar to another study (67.8%) among medical students in India [33]. However, it was lower than estimates (over 75%) among women in the general population, government officials, medical personnel in cervical cancer screening program sites [25], [27], and among a small sample of female college students [28], but much higher than the 36.2% among parents of young adolescents [26], or 40.8% among female sex workers in China [36]. The acceptability of cervical cancer screening was similar to that among women in the general population in a high-incidence area (also a cervical cancer screening program site) in China [29] and lower than the 85% in a small-scale study [37]. The higher acceptability of HPV vaccines and screening in cervical cancer screening program sites may partly be explained by years of educational campaigns as part of government sponsored mass screening programs and thus an increased knowledge level in these areas. Education programs among university students and employed women in mainland China [28] and among adolescents in Hong Kong [38] improved the acceptability of HPV vaccines by about 10%. This emphasizes the importance of targeted education for cervical cancer in school or elsewhere.

Cervical cancer education is relevant also in regards to the poor knowledge of cervical cancer and its prevention. Fewer than 85% of medical students knew the right answer to all of the seven selected knowledge questions in our study. Surprisingly, only 14.4% and 29.0%, respectively, knew that persistent HPV infection was the necessary cause of cervical cancer and that there was a prophylactic HPV vaccine available for cervical cancer in the world. Fewer than 50.0% agreed that cervical cancer could be prevented by HPV vaccines, while 80.0% thought cervical cancer could be prevented by screening. All these daunting statistics point to the lack of necessary education about cervical cancer prevention for medical students in school. In addition, we found that a higher level of knowledge was positively associated with acceptance of HPV vaccines and cervical cancer screening, which is consistent with findings in other studies in mainland China [26], [27], [29], [37]. Since the medical students are future care providers and sources of medical knowledge, their knowledge and attitudes will directly impact the decisions concerning HPV vaccination and cervical cancer screening among patients [39]. Thus, improving medical students' knowledge of cervical cancer appears to be imperative in order to address the barriers to cervical cancer control programs in China.

In addition to the knowledge level, factors such as gender, attitude towards premarital sex, and choice of major subject were important predictors of the acceptability of HPV vaccines and cervical cancer screening. Female medical students were more likely to accept vaccines and screening in our study, which is consistent with the findings for HPV vaccination among health care providers [27]. Since cervical cancer is a female malignancy, it is understandable that females may have better understanding of the disease and thus be more likely to accept prevention. In another study among parents of young adolescents, mothers were more reluctant to accept HPV vaccines for their children because they were more suspicious of newly-developed vaccines [26]. We found that students with negative or uncertain attitudes towards premarital sexual behavior are less likely to accept HPV vaccines or screening compared with those with positive attitudes. This may be explained by the likelihood that the latter students tend to seek out sexual health information and consequently know more about cervical cancer. Since HPV is a sexually transmitted infection, higher acceptance of HPV vaccines and cervical cancer screening among those who favor premarital sex is a good sign for prevention of the infection and cervical cancer. However, another study showed that women with first experience of sexual intercourse after the age of 21 years were more likely to receive screening than those who first had sex at, or before, the age of 20 [30]. This underscores the importance of cervical cancer education in the early period of college or university for medical students. In our study, clinical students more readily accepted cervical cancer than non-clinical medical students, which aligned well with a similar study among medical students in India [33]. Obviously, students in clinical medicine are more exposed to medical courses and other sources of health information.

Perceptions of the timing, target population, and venue for immunization were explored in our study. 35.4% and 46.4%, respectively, thought that HPV vaccines ought to be provided at age 13–18 years, junior or senior middle school or equivalent, which is fairly consistent with the findings in another study among parents of adolescents [26]. The percentage of students who preferred vaccines before sexual debut was 65.1%, much higher than among other population groups in mainland China [25], [26], [40] probably due to the overall better knowledge of cervical cancer among medical students. Over half of the students thought that HPV vaccines can be given to boys. This is a positive signal for the possibility of an HPV vaccination program for men in future in China, because the quadrivalent HPV vaccine for males has been recommended to prevent genital warts and HPV transmission by the Advisory Committee on Immunization Practices in the United States since 2009 [41]. Of course, since there are uncertainties around the evidence of cost-effectiveness for vaccination among males in other countries [42]–[44], it is still early to consider this possibility in China. Consistent with an earlier study [26], local CDC was the most-cited venue for immunization. Although school-based HPV vaccination programs have been applied in other countries, it might add huge administrative costs in China where the CDC network is an existing system for the delivery and management of vaccines [45]. With wide acceptability, the local CDC can be recommended as a venue for HPV vaccination in the future. Over half of students were willing to pay a price lower than 300 RMB (50 USD) for domestic or imported HPV vaccines, and a much higher percentage of students would like to receive or recommend imported vaccines, which might reflect their lack of confidence in the effectiveness and safety of domestic vaccines [46]. Current bivalent and quadrivalent vaccines are close to the end of clinical trial in China, while domestically developed cheaper HPV vaccines are still at early Phase III trial. Current HPV vaccines are expensive (about 400 USD for three doses) considering the average Chinese purchasing power, and they are expected to be more expensive than domestic vaccines. Earlier studies in other countries indicate that the price of HPV vaccines is a barrier to immunization among the population [47]–[49]. In addition, 40.9% of medical students requested price regulation and subsidy for future HPV vaccine programs. In this regard, heavy subsidies for HPV vaccines would be required from the government, or external funding bodies to increase the coverage if they were paid out of pocket in future.

Perceptions of techniques, onset, interval and pricing of cervical cancer screening were assessed in this study. To our surprise, 41.4% of medical students did not know any screening techniques; only 31.6% and 41.1%, respectively, knew the two common techniques, Pap smear and HPV DNA testing. The fact that these two techniques are widely used in clinical practice reflects problems with emphasis on cancer treatment instead of screening and prevention [19] and inadequate education in cervical screening in medical school [50]. The recognition of these problems can be seen in the recent call for cancer screening education in Chinese medical schools [51]. It is recommended that screening should be routinely utilized in women immunized with full doses of HPV vaccines since the vaccines mainly protect against cervical cancer attributable to HPV 16/18 [52]. However, only 72.6% believed that women who have already been vaccinated require cervical screening. Thus education for cervical screening is still relevant in China in an era of HPV vaccination. 41.9% and 32% of medical students thought women should start being screened at 20 years and 25 years, respectively, while 49.2% and 42.1% considered that screening should be given every year and two to four years, respectively. Current guidelines in the US recommend cytology-based screening women aged 21 to 65 years every three years, or for women aged 30 to 65 years who intend to increase the interval, concurrent screening with cytology and HPV DNA testing every five years [6]. In this regard, half of the medical students had an erroneous belief as to the starting age and interval for screening. This might be deeply rooted in the practice in mainland China that many organized health programs such as employment-based physical examination include annual cervical screening [53], partly due to lack of Chinese guidelines for cervical screening. This gap in practice might be reversed through education of medical students in school.

There are certain limitations in the study. Firstly, the medical students surveyed were selected from medical universities or colleges in southwest China. The results may not be readily generalizable to the medical students in other areas, since cities in this part of China are generally less developed economically. However, since students from both cosmopolitan areas such as Chengdu and small cities were sampled, the conclusion may reflect part of the actual medical education scenario. Secondly, since we did not use a standard validation procedure for the questionnaire design, information collected might not be completely representative of actual conditions due to lack of validity and reliability measures. However, we did carefully incorporate input and comments from former staff in the field and participants in pilot surveys in designing the questionnaire, and this transparent procedure can ensure a relatively reliable and valid questionnaire. Thirdly, caution should be taken when interpreting the acceptability of HPV vaccines and cervical cancer screening among medical students. Acceptability may not necessarily mean that they will advise use of vaccines in practice. Knowing something good does not translate into practicing something good. Some factors that might influence their acceptability such as religion and concern for promiscuity are not analyzable in current study. Fourthly, our results only reflect the status quo of current cervical cancer education for medical students. Most of them may increase their knowledge rapidly in health care practice. Finally, constrained by the design, the study might be subject to selection and information bias due to some participants' refusal to participate and data missing in completing the questionnaires by some medical students.

In conclusion, the study indicates low acceptability of HPV vaccines and cervical cancer screening among medical students. The acceptability is associated with relevant knowledge, gender, attitude towards premarital sexual behavior, and the area of major in a course of medical studies. It is important to improve the knowledge of HPV and cervical cancer among medical students in order to supply well-informed health care providers for prevention and control of cervical cancer.

## Supporting Information

Figure S1 Supplementary questionnaire: the questionnaire was used to survey the knowledge and awareness of HPV, cervical cancer, HPV Vaccines, and cervical cancer screening among medical students in Southwest China.
(DOC)
