# Supplementary material for: Acceptability and Correlates of Primary and Secondary Prevention of Cervical Cancer among Medical Students in Southwest China: Implications for Cancer Education
Source: PLoS One. 2014 Oct 31;9(10):e110353. doi: 10.1371/journal.pone.0110353 (PMC4215919; doi:10.1371/journal.pone.0110353)
Supplement: Figure S1 — Supplementary questionnaire: the questionnaire was used to survey the knowledge and awareness of HPV, cervical cancer, HPV Vaccines, and cervical cancer screening among medical students in Southwest China. (DOC) [file pone.0110353.s001.doc]

**Instructions**

Thank you for participating in this **anonymous survey**. All information you provide will be used for **research purposes** only. **Most questions require single response, those requiring multiple responses are clarified in parentheses.**

Questionnaire ID:

Date (month/day): | | |-| | |

**Section 1 Basic information**

- 1. **Date of Birth: ______ (Y) ____ (M)**
  2. **Gender:** 口1=Male 口2=Female
  3. **Ethnicity:** 口1=Han 口2=Other, please comment
  4. Grade: 口1=1 口2=2 口3=3

口4=4 口5=5

1.5 **Major：** 口1=Clinical medicine

口2=Dentistry 口3=Preclinical medicine

口4=Forensic medicine 口5=Pharmacy

口6=Preventive medicine 口7=Rehabilitation

口8=Nursing 口9=Medical

口10= Clinical laboratory science

口11=Management in hospitals and pharmaceutical enterprises 口12=Other

1.6 **Clinical Internship：**口Yes（ months）

口No

**Section 2 Knowledge and Awareness**

**I. Knowledge and awareness of HPV and Cervical Cancer**

2.1 Have you ever heard of human papillomavirus (HPV)?

口1=Yes 口2=No

2.2 Is HPV sexually transmitted?

口1=Yes 口2=No 口3=I don’t know

2.3 Is HPV infection common among women?

口1=Yes 口2=No 口3=I don’t know

2.4 Can most HPV infections be symptomatic?

口1=Yes 口2=No 口3=I don’t know

2.5 Can HPV infect a man?

口1=Yes 口2=No 口3=I don’t know

2.6 Can HPV be cleared by the immune system?

口1=Yes 口2=No 口3=I don’t know

2.7 Is treatment necessary for HPV infection?

口1=Yes 口2=No 口3=I don’t know

2.8 Can HPV infection cause cervical cancer?

口1=Yes 口2=No 口3=I don’t know

2.9 Can HPV cause genital warts?

口1=Yes 口2=No 口3=I don’t know

2.10 Can some HPV genotypes cause **both** cervical cancer and genital warts?

口1=Yes 口2=No 口3=I don’t know

2.11 Is persistent HPV infection the necessary cause of cervical cancer?

口1=Yes 口2=No 口3=I don’t know

2.12 Can cervical cancer be cured?

口1=No 口2=Only precursor lesions

口3=Only early cancer or precursor lesions

口4=I don’t know

**II. Knowledge and Awareness of HPV Vaccines**

2.13 Can cervical cancer be prevented by HPV vaccines?

口1=Yes 口2=No 口3=I don’t know

2.14 Is there any HPV vaccine available for cervical cancer, penile cancer, anal cancer, and genital warts, globally?

口1=Yes 口2=No 口3=I don’t know

2.15 Is there any HPV vaccine available in China?

口1=Yes 口2=No 口3=I don’t know

2.16 To which age group should HPV vaccines be given?

口1=0-12 years 口2=13-18 years

口3=19-25 years 口4=26 years or above

口5=I don’t know

2.17 Can HPV vaccines be given to boys?

口1=Yes 口2=No 口3=I don’t know

2.18 Which is the most appropriate stage for HPV vaccination?

口1= Before sexual debut

口2= After sexual debut

口3=I don’t know

2.19 Can HPV vaccines be given to a woman already infected with HPV infection?

口1=Yes 口2=No 口3=I don’t know

2.20 Can HPV vaccines be given to a sexually active woman?

口1=Yes 口2=No 口3=I don’t know

2.21 Do girls/women need to be screened for HPV before getting vaccinated?

口1=Yes 口2=No 口3=I don’t know

2.22 How many doses of HPV vaccines are required for protection?

口1=1 口2=2 口3=3

口4= I don’t know

2.23 Is it safe to have multiple sexual partners after full course of HPV vaccines?

口1=Yes 口2=No 口3=I don’t know

2.24 Do women who have already been vaccinated require cervical cancer screening?

口1=Yes 口2=No 口3=I don’t know

2.25 How much protection does HPV vaccines provide against cervical cancer?

口1=100% 口2=90% 口3=70%

口4=I don’t know

2.26 What are your sources of knowledge about HPV vaccination (multiple responses)?

口1=School courses 口2=Hospital

口3=Family / friends

口4= Internet / television

口5=Publication

口6=Other, please comment

2.27 Would you like to receive or recommend HPV vaccination?

口1=Yes (please jump to 2.29)

口2=No

口3=I don’t know (please jump to 2.29)

2.28 What do you think will be **the most important obstacle** preventing yourself from receiving or recommending HPV vaccination?

口1=High cost

口2=Concern about complications

口3=Concern about efficacy of vaccines

口4=Inadequate information

2.29 Has anybody (family/friends) sought your opinion regarding HPV vaccination?

口1=Yes 口2=No

2.30 Would you like to receive more HPV related education by experts?

口1=Yes 口2=No

2.31 Which venue is the most appropriate for HPV vaccination (multiple responses)?

口1=Local CDC
 口2=Community health center/local clinic

口3= Women and children's hospital

口4= General hospital

口5=School

口6=I don’t know

2.32 Which education stage is most appropriate for HPV vaccination?

口1=Primary school

口2=Junior high school

口3=Senior high school (junior technical school or vocational school)

口4=University (senior technical school)

口9=I don’t know

2.33 **How much** are you willing to pay for **imported HPV vaccines** (current price: about 2400 RMB)?

口1=Below 100 RMB 口2=100-300 RMB 口3=300-500R MB 口4=500-1000 RMB

口5=1000-2400RMB

2.34 **How much** are you willing to pay for **domestic HPV vaccines**?

口1=Below 100 RMB 口2=100-300 RMB 口3=300-500 RMB 口4=500-1000 RMB

口5=1000-2400 RMB

2.35 Which vaccines would you prefer to receive or recommend?

口1=Domestic HPV vaccines

口2=Imported HPV vaccines

口3=Either, dependent on price

2.36 What is your attitude toward a future HPV vaccination program in China (multiple responses)?

口1=Positive, since the vaccines can prevent cervical cancer/genital warts

口2=Positive, but requesting pricing regulation and subsidy by | |%

口3=Neutral, since the price will be high and the consumption capacity of ordinary Chinese should be considered.

口4=Neutral, since long-term efficacy and side effects should be evaluated.

口5=Negative, since it may lead to promiscuity.

口6=Other, please comment

**III Knowledge and Awareness of Cervical Cancer Screening**

2.37 Can cervical cancer be prevented by screening?

口1=Yes 口2=No 口3=I don’t know

2.38 What techniques are you aware of for cervical cancer screening (multiple responses)?

口1= Pap smear 口2=HPV DNA

口3= VIA/VILI 口4= I don’t know

2.39 How often should women receive cervical cancer screening?

口1=Every 1 year 口2=Every 2-4 years

口3=Every 5-9 years

口4=Every 10 years

2.40 When should women start screening?

口1=20 years 口2=25 years

口3=30 years 口1=35 years

口5= I don’t know

口6=Other, please comment

2.41 Would you like to receive or recommend cervical cancer screening?

口1=Yes (please jump to 2.43)

口2=No

口3=I don’t know (please jump to 2.43)

2.42 What do you think will be **the most important obstacle** preventing yourself from receiving or recommending cervical cancer screening?

口1=High cost 口2=Concern about complications

口3=Concern about effectiveness

口4=Inadequate information

2.43 How much do you think is reasonable for cervical cancer screening for one occasion (RMB)?

口1=Below 10 RMB 口2=10-49 RMB

口3=50-99 RMB 口4=100-149 RMB

口5=150-199 RMB

口6=200 RMB or above

2.44 Has anybody (family/friends) sought your opinion regarding cervical cancer screening?

口1=Yes 口2=No

2.45 Would you like to receive more education on cervical screening by experts?

口1=Yes 口2=No

2.46 What are your sources of knowledge about cervical cancer screening (multiple responses) ?

口1=School courses 口2=Hospital

口3=Family / friends

口4= Internet / television

口5=Publication

口6=Other, please comment

**Section 3 Sexual Behavior and Attitude**

3.1 The age of your sexual maturity (years)

| | |

3.2 What is your attitude towards premarital sexual behavior?

口1=Positive 口2=Negative

口3=Neutral 口4=Undecided

3.3 Have you practiced sexual behavior?

口1=Yes

口2=No **(the survey ends if you choose No)**

3.4 The age of your first sexual behavior (years) | |, and the number of sexual partners you have | | (fill in 00 if you decline to answer).

3.5 How often do you or your partner use condom when you have sex?

口1= Every time 口2= Often

口3= Sometimes 口 4= Never

3.6 Why do you use condom?

口To prevent pregnancy

口To prevent HIV/AIDS

口 Other, please comment
